# The Child-care Food and Activity Practices Questionnaire (CFAPQ): development and first validation steps

**DOI:** 10.1017/S1368980015003444

**Published:** 2015-12-04

**Authors:** Jessica S Gubbels, Ester FC Sleddens, Lieke CH Raaijmakers, Judith M Gies, Stef PJ Kremers

**Affiliations:** NUTRIM School for Nutrition and Translational Research in Metabolism, Department of Health Promotion, Maastricht University, PO Box 616, 6200 MD Maastricht, The Netherlands

**Keywords:** Child care, Comprehensive Feeding Practices Questionnaire, Day care, Feeding, Nutrition, Parenting practices, Physical activity, Preschooler Physical Activity Parenting Practices

## Abstract

**Objective:**

To develop and validate a questionnaire to measure food-related and activity-related practices of child-care staff, based on existing, validated parenting practices questionnaires.

**Design:**

A selection of items from the Comprehensive Feeding Practices Questionnaire (CFPQ) and the Preschooler Physical Activity Parenting Practices (PPAPP) questionnaire was made to include items most suitable for the child-care setting. The converted questionnaire was pre-tested among child-care staff during cognitive interviews and pilot-tested among a larger sample of child-care staff. Factor analyses with Varimax rotation and internal consistencies were used to examine the scales. Spearman correlations, *t* tests and ANOVA were used to examine associations between the scales and staff’s background characteristics (e.g. years of experience, gender).

**Setting:**

Child-care centres in the Netherlands.

**Subjects:**

The qualitative pre-test included ten child-care staff members. The quantitative pilot test included 178 child-care staff members.

**Results:**

The new questionnaire, the Child-care Food and Activity Practices Questionnaire (CFAPQ), consists of sixty-three items (forty food-related and twenty-three activity-related items), divided over twelve scales (seven food-related and five activity-related scales). The CFAPQ scales are to a large extent similar to the original CFPQ and PPAPP scales. The CFAPQ scales show sufficient internal consistency with Cronbach’s *α* ranging between 0·53 and 0·96, and average corrected item–total correlations within acceptable ranges (0·30–0·89). Several of the scales were significantly associated with child-care staff’s background characteristics.

**Conclusions:**

Scale psychometrics of the CFAPQ indicate it is a valid questionnaire that assesses child-care staff’s practices related to both food and activities.

Energy balance-related habits (i.e. dietary intake, physical activity and sedentary behaviour) originate in early childhood and track into later life^(^ ^1^ ^,^ ^2^ ^)^. Parents can have a strong influence on these habits by the parenting practices they use^(^ ^3^ ^)^. Parenting practices are content-specific acts of parenting^(^ ^4^ ^)^, and include setting rules about dietary intake or activity behaviour, acting as a role model, and educating children about food and physical activity^(^ ^5^ ^–^ ^8^ ^)^.

Various reviews have summarized the many studies regarding the influence of parenting practices on children’s dietary intake (e.g. references 9 and 10) and physical activity and sedentary behaviour (e.g. reference 11). With regard to food-related parenting practices^(^ ^9^ ^)^, instrumental and emotional feeding, in which food is used to change children’s behaviour or mood, seem less effective or sometimes show undesirable effects. Stimulating healthy intake seems to be a more promising approach. Also with regard to physical activity^(^ ^11^ ^)^, supportive and encouraging parenting practices seem to hold the most promising results. In line with the growing number of studies examining energy balance-related parenting, there are also numerous instruments to assess food-related^(^ ^12^ ^)^ and activity-related^(^ ^11^ ^,^ ^13^ ^,^ ^14^ ^)^ parenting, including various validated instruments.

However, an increasing number of children are attending non-parental child care for one or more days per week; more than half of European toddlers attend child-care or pre-school education facilities^(^ ^15^ ^)^. In view of the increasing child-care use, various authors have called for increased attention to the influence of child-care staff practices on children’s energy balance-related behaviour^(^ ^16^ ^–^ ^19^ ^)^. Child-care use is associated with an increased overweight risk throughout childhood^(^ ^20^ ^,^ ^21^ ^)^. Moreover, differences between children’s dietary intake, physical activity and sedentary behaviour can be partially attributed to the child-care centre or the pre-school the child is attending^(^ ^22^ ^,^ ^23^ ^)^. However, a limited number of studies have examined the association between child-care practices and children’s nutrition. For instance, we recently showed^(^ ^24^ ^)^ that children ate more vegetables when child-care staff encouraged them. Children ate more fruit and less sweet snacks when staff involved them in food preparation^(^ ^24^ ^)^. Other studies have shown beneficial effects of staff modelling healthy food intake or talking about healthy food^(^ ^25^ ^)^, or combining these practices by using enthusiastic modelling, in which staff verbally confirmed that the food they tasted, tasted good^(^ ^26^ ^)^. As regards physical activity, encouragement of physical activity by staff has been linked to increased activity levels^(^ ^19^ ^,^ ^27^ ^)^. Findings with regard to child-care staff’s initiation of and participation in play are mixed^(^ ^27^ ^,^ ^28^ ^)^.

A recent review of physical activity and healthy eating environmental audit tools in youth care settings indicated that there are a handful of audit tools for the child-care setting^(^ ^29^ ^)^. However, most of these instruments focus mainly on the physical child-care environment or child-care policies. Staff’s food-related and activity-related practices are included in some instruments, although this concerns only a limited selection of practices (e.g. references 30 and 31). Hughes *et al*. have developed a questionnaire to assess child-care staff’s feeding styles, which are conceptually different from specific food-related practices^(^ ^32^ ^)^. In addition, Dev and colleagues have used parenting practices instruments to assess child-care staff’s food-related practices^(^ ^33^ ^,^ ^34^ ^)^, although these were not validated for the child-care setting. To our knowledge, validated questionnaires to specifically assess the broad range of child-care staff’s food-related and activity-related practices in detail are not yet available.

The lack of such questionnaires to specifically assess child-care staff practices is a large contrast to the abundance of questionnaires to assess parenting practices and reflects the gap between our knowledge regarding practices in both settings (home and child care)^(^ ^24^ ^)^. Therefore, the aim of the current study was to develop and validate a questionnaire to measure food-related and activity-related practices of child-care staff, based on existing, validated parenting practices questionnaires.

## Methods

### Questionnaire selection

Two parenting practices questionnaires were selected for conversion to the child-care setting: the Comprehensive Feeding Practices Questionnaire (CFPQ) of Musher-Eizenman and Holub^(^ ^35^ ^)^ assessing food-related parenting practices; and the Preschooler Physical Activity Parenting Practices (PPAPP) questionnaire of O’Connor *et al*.^(^ ^36^ ^)^ assessing activity-related practices. The selection was done by three of the authors of the current paper (J.S.G., E.F.C.S. and S.P.J.K.), who are experts in the field of parenting and/or child care. The questionnaires were selected based on previous studies using parenting practices instruments in the child-care setting^(^ ^33^ ^,^ ^34^ ^)^, the findings of recent reviews of parenting practices instruments^(^ ^11^ ^–^ ^14^ ^)^ and an additional literature review to find any questionnaires published after these reviews. Criteria for the selection of the questionnaires were the validity and suitability of the questionnaire for the age group (0–4 years old) and the suitability of the item content for translation to the child-care setting. All decisions were made through extensive author review meetings.

The CFPQ has been previously used to assess child-care staff’s food-related practices in the child-care setting by Dev and colleagues^(^ ^33^ ^,^ ^34^ ^)^. However, they applied the original scales of the parenting version^(^ ^35^ ^)^ to their child-care version^(^ ^33^ ^,^ ^34^ ^)^. Building on the work of Dev *et al*. and the considerations above, we decided to select the CFPQ for the current validation study. The CFPQ is partly based on one of the most widely used scales in the child feeding literature: the Child Feeding Questionnaire (CFQ), developed by Birch *et al*.^(^ ^37^ ^)^. However, because the CFQ did not fully capture the range of food-related practices, the CFPQ was developed^(^ ^35^ ^)^. The CFPQ assesses food parenting practices using forty-nine items divided over twelve scales (see online supplementary material, Supplemental Table 1): Child control, Emotion regulation, Encourage balance and variety, Environment, Food as reward, Involvement, Modelling, Monitoring, Pressure to eat, Restriction for health, Restriction for weight control and Teaching about nutrition. All items are answered using a 5-point Likert scale ranging from ‘never’ to ‘always’ for the questions and from ‘disagree’ to ‘agree’ for the statements. The validation studies of the CFPQ show good fit of the final model, with Cronbach’s *α* ranging from 0·58 to 0·81^(^ ^35^ ^)^. The items of the CFPQ were independently translated to Dutch by four of the authors (J.S.G., E.F.C.S., L.C.H.R. and J.M.G.). In the case of disagreement, the fifth author (S.P.J.K.) was involved to decide on the final item.

The PPAPP measures activity-related parenting practices using thirty-two items on two main scales: Encouraging physical activity (seventeen items) and Discouraging physical activity (fifteen items)^(^ ^36^ ^)^ (see online supplementary material, Supplemental Table 2). The Encouraging and Discouraging scales are split up further in various subscales and single items. The Encouraging scale includes the Engagement subscale and two single items. The Discouraging scale consists of four subscales: Promote inactive transport, Promote screen time, Psychological control and Restriction for safety concern. Answers are on a 5-point Likert scale ranging from ‘never’ to ‘always’. Validation studies of the questionnaire showed a test–retest reliability ranging from 0·56 to 0·85, and Cronbach’s *α* values between 0·50 and 0·90^(^ ^36^ ^,^ ^38^ ^)^. A Dutch translation of the PPAPP was already available from a previous study^(^ ^39^ ^)^. The translation procedure for the PPAPP was in line with that of the CFPQ, with multiple experts independently translating the questionnaire.

### Questionnaire conversion

After the CFPQ and PPAPP were selected, their items were reviewed by the present authors for applicability in the child-care setting. Several items were dropped because they were not applicable for the child-care setting. For the CFPQ, four items were dropped (see online supplementary material, Supplemental Table 1), leaving forty-five items. For instance, ‘I encourage my child to participate in grocery shopping’ from the Involvement scale was dropped, as children do not participate in grocery shopping in child-care. For the PPAPP, five items were dropped (see Supplemental Table 2), leaving twenty-seven items. An example of an item from the PPAPP that was not applicable was ‘How often do you take your child to sport practice or game in which he/she is enrolled?’

The remaining items were translated to the child-care setting. In line with the translation to Dutch, the items were independently translated to the child-care setting by four of the authors (J.S.G., E.F.C.S., L.C.H.R. and J.M.G.). In the case of disagreement, the fifth author (S.P.J.K.) was involved to decide on the final item. For most items, the conversion to the child-care setting simply meant replacing ‘my child’ by ‘the children’. For instance, ‘I show my child how much I enjoy eating healthy foods’ was converted to ‘I show the children how much I enjoy eating healthy foods’. For other items, additional changes had to be made. For instance, ‘How often do you play a sport or active game together as a family?’ was changed to ‘How often do you play a sport or active game together with the children (and perhaps with other child-care staff)?’

### Qualitative pre-test

To pre-test the developed questionnaires, child-care staff were approached to participate in a cognitive interview. The aim of this cognitive interview was to find errors in the questionnaire or unsuitable items, including unclear instructions or answer options, that could influence the outcome of the questions^(^ ^40^ ^)^. For the interviews, thirteen child-care centres in the Netherlands were contacted via telephone. Four child-care centres agreed to participate and a total of ten child-care staff members from these four centres were interviewed. The participants were all female and their age ranged between 20 and 55 years.

First, the researchers explained the procedure to the participants. During the interviews, participants were asked to indicate good questions with ‘+’, questions that can be improved with ‘–’, and with ‘0’ when they did not have an opinion. When participants indicated a question with ‘+’, it was discussed what was good about the question. When participants rated a question with ‘–’, it was discussed what could be improved about the question. Next, there were some general questions about the clarity of the instructions and the answer options of the items. Subsequently, the applicability of some specific items was discussed.

The interviews were recorded on a voice-recording device. Additionally, the interviewers took field notes. Based on the outcomes of the interviews, a few additional changes had to be made to the items. These were mostly minor changes in wording or sentence structure. In some cases, examples were added to the items for clarification. Furthermore, several additional items were dropped based on the responses of the interview participants. These were five items of the CFPQ and four items of the PPAPP (see online supplementary material, Supplemental Tables 1 and 2). For the CFPQ, five items of the Restriction for weight control scale were deleted, as the child-care staff could not relate to these questions; weight control was beyond their influence and beyond their responsibility. Another example was that all child-care staff indicated that they had an enclosed and locked playground. The PPAPP questions regarding not letting children play outside because of worries about traffic, crime or strangers thus were not relevant. Supplemental Tables 1 and 2 show which items were deleted at this stage.

The combined and converted pilot-test version of the questionnaire, named the Child-care Food and Activity Practices Questionnaire (CFAPQ), contained sixty-three items: forty regarding diet and twenty-three regarding physical activity. The items included in the CFAPQ are shown in online supplementary material, Supplemental Tables 1 and 2.

### Procedure of pilot test

The pilot-test version of the questionnaire was pilot-tested among a larger sample of child-care staff. In addition to the CFAPQ items, several items were added regarding participants’ demographics. These were the child-care staff’s age (18–25 years, 26–35 years, 36–45 years, 46–55 years, 56–65 years, >65 years), gender, weight (in kilograms), height (in centimetres), educational level (child-care staff education (in Dutch: Sociaal Pedagogisch Werker) level 3, 4 or 5), whether they had children themselves, total number of groups in the child-care centre in which they were working at the time of the questionnaire, number of years working in the child-care centre in which they were working at the time of the questionnaire, and total number of years working in the child-care setting in general.

A total of 1028 randomly selected child-care centres were approached, mostly via email or telephone. Email addresses and telephone numbers were obtained from a Dutch national database of child-care centres and pre-schools (in Dutch: Landelijk Register Kinderopvang en Peuterspeelzalen^(^ ^41^ ^)^). Child-care centres had the ability to complete the questionnaire online^(^ ^42^ ^)^ or to request a paper version, which was sent to them with a prepaid return envelope. Participants were recruited during April 2014. Data collection took place during April and May 2014. In total, 256 child-care staff members agreed to participate. It is unknown whether more than one staff member participated per child-care centre. Of the 256 participants, 178 (69·5 %) completed at least part of the questionnaire and were retained for further analyses.

### Statistical analyses

Statistical analyses were performed using the statistical software package IBM SPSS Statistics 21. Descriptive statistics were used to describe the background characteristics of the sample. Both internal reliability coefficients (Cronbach’s *α*) and corrected item–total correlations (CITC) were calculated. A cut-off point of 0·50 for Cronbach’s *α* was used^(^ ^43^ ^)^. CITC values above 0·30 were regarded as ‘good’ and values below 0·15 as ‘unreliable’^(^ ^44^ ^)^.

To check whether the data were adequate for factor analysis, the Kaiser–Meyer–Olkin measure of sampling adequacy and Bartlett’s test of sphericity were measured. A Kaiser–Meyer–Olkin value of 0·5 was considered suitable. Furthermore, the Bartlett’s test of sphericity needed to be significant^(^ ^44^ ^)^. A principal components analysis with Varimax rotation was performed on items of the CFAPQ (the forty items of the food-related practices and twenty-three of the activity-related practices part separately) to determine whether the original factor structure would be replicated in this sample. All factors had to have an eigenvalue above 1·0. The scree plot was used to determine the number of factors^(^ ^45^ ^,^ ^46^ ^)^. The cut-off point for factor loadings was 0·30^(^ ^45^ ^)^. In case the factor structure did not equal the factor structures of the original questionnaires, we forced to retrieve fewer factors to potentially improve the interpretability of the results.

In addition, the correlations between the scales based on the factor solutions were examined using Pearson correlations. Correlations between the scales and child-care staff’s background characteristics were examined using independent-samples *t* tests for bivariate variables (i.e. gender, whether they had own children and educational level), ANOVA for categorical variables (i.e. age, years of experience in current centre and in general) and Spearman correlations for continuous variables (i.e. BMI and number of groups in current centre).

## Results

### Background characteristics of the participants of the pilot test

A total of 178 child-care staff members filled out the questionnaire. Table 1 shows their background characteristics. The vast majority of the participants were female (98·9 %). The largest age group was 26–35 years (42·7 %). Most had a normal weight (70·4 %) and did have children themselves (65·2 %). Most participants worked in a mixed age group (53·7 %) and had been working at their current child-care centre for less than 5 years (44·4 %).

**Table 1:** Background characteristics of participants in the pilot test: child-care staff members (*n* 178), the Netherlands, April–May 2014

	*n*	%	Mean	sd
Gender				
Female	176	98·9		
Male	2	1·1		
Age group				
18–25 years	15	8·4		
26–35 years	76	42·7		
36–45 years	36	20·2		
46–55 years	35	19·7		
56–65 years	16	9·0		
BMI (kg/m^2^)			23·9	2·2
Normal weight (20–25)	114	70·4		
Overweight (25–30)	38	23·5		
Obese (>30)	10	6·2		
Education†				
Child-care staff level 3	39	22·4		
Child-care staff level 4	63	36·4		
Child-care staff level 5	88	50·9		
Number of own children				
None	62	34·8		
One or more	116	65·2		
Type of group†				
Baby group (0–1 years)	24	13·6		
Toddler group (2–4 years)	43	24·3		
Mixed age group (0–4 years)	95	53·7		
Other type of group	39	22·0		
Total experience in child-care				
<5 years	37	20·8		
6–10 years	51	28·7		
11–15 years	40	22·5		
>15 years	50	28·1		
Experience in the current centre				
<5 years	79	44·4		
6–10 years	43	24·2		
11–15 years	27	15·2		
>15 years	29	16·3		
Total number of groups at current centre			3·6	2·2

*n* deviates from total sample size due to missing values. Percentages presented in the table represent valid percentages.

† Multiple answers possible.

### Factor analyses and psychometric evaluation of the CFAPQ

The online supplementary material, Supplemental Tables 3 and 4 show the frequencies and means of the answers to the singe items. The data were considered appropriate for factor analysis, since the Kaiser–Meyer–Olkin measure of sampling adequacy was 0·66 for the food-related practices items and 0·78 for the activity-related practices items. The Bartlett’s test of sphericity was significant for both parts of the questionnaire at a level of <0·001.

#### Food-related practices

The factor analysis was based on forty items. As we repeatedly found that one item belonging to the scale of Encouraging balance and variety (‘Do you encourage the children to eat healthy foods before unhealthy ones?’) did not load on the intended factor, we decided to delete this item from the factor analysis. However, we decided to retain this item for further analyses as a single item. Factor analysis on the remaining thirty-nine items resulted in a twelve-factor solution. The six items of the scale of Restriction had multiple cross-loadings on several factors, even after forcing the factor solution to retrieve fewer factors. We therefore deleted the full scale of restriction and performed a separate factor analysis on the six items conceptually belonging to Restriction. Even though a two-factor solution was found, we forced to a one-factor solution and the findings were acceptable (see Table 2). The factor loadings ranged from 0·43 to 0·71, and internal consistency was adequate (Cronbach’s *α*=0·60 and the CITC ranged from 0·23 to 0·44 for the six items).

**Table 2:** Factor structure of the Restriction scale of the food-related practices items of the Child-care Food and Activity Parenting Questionnaire (CFAPQ), percentage of variance accounted for by each factor and reliability estimates

Food-related practices items: Restriction	Factor 1	Factor 2	Forced to one factor
If I did not guide or regulate the children’s eating, they would eat too many junk foods		0·71	**0**·**58**
I have to be sure that the children do not eat too much of their favourite products	0·38	0·52	**0**·**71**
I have to be sure that the children do not eat too many sweets (for example, candy, ice cream, cookies or pastries)	0·72		**0**·**65**
I have to be sure that the children do not eat too many high-fat foods (for example, cheese, sausage, cookies)	0·75		**0**·**47**
If a child eats more than usual at a one meal, I try to restrict his/her eating at the next meal		0·78	**0**·**43**
If I did not guide or regulate the children’s eating, they would eat too much of their favourite foods	0·63		**0**·**62**
Percentage of variance accounted for	34·01	19·25	34·01
Cronbach’s *α* coefficient			0·60
Average corrected item–total correlation			0·34
Range corrected item-total correlation			0·23–0·44
Mean			3·37
sd			0·63

*n* 159. Only factor loadings higher than the absolute value of 0·30 are reported. Bold values represent the final factor structure.

Factor analysis on the remaining thirty-three items retrieved a nine-factor solution with eigenvalues higher than 1, accounting for 64·68 % of the total variance. All except one (the scale of Food as a reward) of the original scales of the CFPQ^(^ ^35^ ^)^ did load on the intended factors. When looking at the rotated factor solution, the three items conceptually belonging to the scale of Food as a reward had high factor loadings on multiple factors. We therefore forced the factor analyses to retrieve an eight-factor solution, accounting for 61·12 % of the total variance. In line with the nine-factor solution, cross-loadings (>0·30) were found for the items of the scale Food as a reward on multiple factors. Finally, we forced to a seven-factor solution. This factor solution accounted for 57·35 % of the total variance with eigenvalues higher than 1. The findings of this factor solution are presented in Table 3. Most of the scale items loaded as expected, conforming to the study of Musher-Eizenman and Holub^(^ ^35^ ^)^. Three factors retrieved contained two scales: factor 2 was represented by the scale of Encourage balance and variety and the scale of Modelling; factor 3 was represented by the scales of Environment and Involvement; and factor 7 was represented by the scales of Emotion regulation and Food as a reward. The other factors contained a single scale of the original constructed scale: Monitoring, Teaching about nutrition, Pressure to eat and Child control. Most items loaded onto a single factor. A few had loadings higher than the absolute value of 0·30 on two and sometimes three factors. These items were allocated to the factor where the theoretical fit was best, conforming to the original factor structure. For two items, one of the scale of Encourage balance and variety and one of the scale of Child control, the factor loading was below 0·30. These items were retained in the final factor solution to provide better comparability with the original factor structure of the CFPQ.

**Table 3:** Factor structure of the food-related practices items of the Child-care Food and Activity Parenting Questionnaire (CFAPQ), percentage of variance accounted for by each factor and reliability estimates

Food-related practices items	F-MON	F-MOD/ENC	F-INV/ENV	F-TN	F-PE	F-CC	F-ER/FR
How much do you keep track of the sweets that the children eat (for example, candy, ice cream, cookies)?	**0**·**92**						
How much do you keep track of the snack food that the children eat (for example, salty crackers, potato chips, cheese puffs)?	**0**·**94**						
How much do you keep track of the high-fat foods that the children eat (for example, cheese, sausage, cookies)?	**0**·**90**						
How much do you keep track of the sugary drinks that the children drink (for example, lemonade, chocolate milk, fruit drink)?	**0**·**89**						
I model healthy eating for the children by eating healthy foods myself		**0**·**72**					
I try to eat healthy foods in front of the children, even if they are not my favourite		**0**·**66**					
I try to show enthusiasm about eating healthy foods		**0**·**82**					
I show the children how much I enjoy eating healthy foods		**0**·**81**					
I encourage the children to try new foods		**0**·**26**†		0·63			
I tell the children that healthy food tastes good		**0**·**47**		0·57			
I encourage the children to eat a variety of foods		**0**·**64**					−0·31
I allow the children to help prepare meals (for example, set the table, prepare sandwiches, etc.)			**0**·**49**				
Most of the food at the child-care centre is healthy			**0**·**62**				
There are a lot of snack foods present in the child-care centre (for example, crackers, potato chips, cheese puffs). **R**			**0**·**68**				
A variety of healthy foods are available to the children at each meal served at the child-care centre			**0**·**77**				
There are a lot of sweets present at the child-care centre (for example, cookies, candy, ice cream). **R**			**0**·**69**				
I discuss with the children why it’s important to eat healthy foods		0·50		**0**·**62**			
I discuss with the children the nutritional value of foods				**0**·**49**			0·33
I tell the children what to eat and what not to eat without explanation. **R**				**0**·**66**			
The children should always eat all of the food on their plate					**0**·**63**		
If a child says ‘I’m not hungry’, I try to get him/her to eat anyway					**0**·**66**		
If a child eats only a small helping, I try to get him/her to eat more					**0**·**65**		
When a child says that he/she is finished eating, I try to get the child to eat another bite of food					**0**·**63**		
Do you let the children eat whatever they want?						**0**·**68**	
At meals, do you let the children choose the foods they want from what is served (for example, choose the bread toppings during lunch)?			0·43			**0**·**49**	−0·33
If the children don’t like the food that is being served, do you make something else?						**0**·**66**	
Do you allow the children to eat snacks whenever they want?						**0**·**59**	
Do you allow the children to leave the table when they are full, even when the other children are not done eating?					−0·52	**0**·**20** †	
When a child gets fussy, is giving him/her something to eat the first thing you do?						0·30	**0**·**56**
Do you give a child something to eat or drink if s/he is upset, even if you think s/he is not hungry?							**0**·**69**
I offer sweets to the children as a reward for good behaviour (for example, cookies, candy, ice cream)			−0·32	−0·39			**0**·**34**
I withhold sweets from the children in response to bad behaviour							**0**·**60**
I offer the children their favourite foods in exchange for good behaviour				−0·63		0·32	**0**·**33**
							
Percentage of variance accounted for	16·68	10·19	9·02	6·55	5·71	4·96	4·23
Cronbach’s *α* coefficient	0·96	0·82	0·67	0·53	0·64	0·54	0·56
Average corrected item–total correlation (for each scale)	0·89	0·57	0·42	0·36	0·42	0·30	0·34
Range corrected item–total correlation (for the items of each scale)	0·86–0·91	0·37–0·70	0·17–0·58	0·19–0·51	0·40–0·43	0·23–0·40	0·14–0·49
Mean	4·20	4·26	4·40	3·55	2·81	2·49	1·29
sd	0·93	0·62	0·60	0·78	0·79	0·55	0·40

*n* 111. Factors are labelled as follows: F-MON, food-related Monitoring; F-MOD/ENC, food-related Modelling/Encourage balance and variety; F-INV/ENV, food-related Involvement/Environment; F-TN, food-related Teaching about nutrition; F-PE, food-related Pressure to eat; F-CC, food-related Child control; F-ER/FR, food-related Emotion regulation/Food as reward. Only factor loadings higher than the absolute value of 0·30 are reported. Sample size used to measure internal consistency estimates: F-MON, *n* 125; F-MOD/ENC, *n* 158; F-INV/ENV, *n* 159; F-TN, *n* 158; F-PE, *n* 159; F-CC, *n* 140; F-ER/FR, *n* 155. The following item was not included in the factor analyses, but retained as a single item: ‘Do you encourage the children to eat healthy foods before unhealthy ones?’ (mean=4·28, sd=1·19). **R=**reverse coded. Bold values represent the final factor structure.

† Although the factor loading was below 0·30, it is depicted in the table and on theoretical grounds the item has provisionally been retained on this factor.

The internal consistency coefficients for the scales are displayed in Table 3 and can be considered adequate, ranging from 0·53 to 0·96. The average CITC are also within acceptable ranges (0·30–0·89). In addition, Tables 2 and 3 show the means and standard deviations of the food-related CFAPQ scales.

#### Activity-related practices

The factor analysis was based on twenty-two items, instead of twenty-three items. We excluded the item ‘How often do you have outdoor toys available for the children (for example, skipping ropes, balls, etc.)?’, as this was the only item representing availability of physical activity materials and therefore did not represent a certain type of practice or behaviour a child-care staff member can perform. The factor analysis revealed a seven-factor solution with eigenvalues higher than 1, accounting for 68·25 % of the total variance. When looking at the rotated factor solution, the three items on the Promote screen time scale (Discouragement) clustered together with factor loadings ranging from 0·54 to 0·86. However, the five items on the Psychological control scale (Discouragement) had high loadings on two factors. In addition, items on the scale of Encouragement had high loading on four factors. Three factors represented the following constructs conceptually belonging to Encouragement of physical activity, namely Modelling (factor 1), Teaching/autonomy support (factor 2) and Going outdoors (factor 5). The item ‘How often do you dance with the children?’ was the only item loading on the fourth factor of Encouragement, factor 7 (factor loading of 0·70), although this item also loaded on factor 1 (Encouragement, Modelling) with a factor loading of 0·28. As this item conceptually belongs to ‘Modelling’, we forced the factor analyses to a six-factor solution.

The six-factor solution accounted for 63·52 % of the total variance with eigenvalues higher than 1. In agreement with the seven-factor solution, the five items on the Psychological control scale (Discouragement) still had high loadings on two factors. However, the item ‘How often do you dance with the children?’ now loaded onto the first (Modelling) and second factor (Teaching/autonomy support) belonging to Encouragement, with a factor loading of 0·32 and 0·33, respectively. We did again force the factor analyses, but now to a five-factor solution.

The five-factor solution explained 58·29 % of the variance in responses of the physical activity practices part of the CFAPQ, with eigenvalues higher than 1. The findings of this factor solution are presented in Table 4. With regard to the Discouragement scale, we perfectly found the two scales of Psychological control (factor 2) and Promote screen time (factor 3). All items of the two scales had the highest factor loading on the factor the item belongs to. With regard to the Encouragement scale, three sub-scales were created, one containing eight items representing Modelling of physical activity (factor 1), another containing five items representing Teaching/autonomy support of physical activity (factor 4), and the final factor containing two items representing Going outdoors (factor 5). Some items of factor 4 had higher factor loadings on factor 1, but were retained to factor 4 as the theoretical fit was best on this factor (i.e. the items ‘How often do you say positive things to motivate children to be more active?’ and ‘How often do you teach the children new and different ways to be active?’).

**Table 4:** Factor structure of the activity-related practices items of the Child-care Food and Activity Parenting Questionnaire (CFAPQ), percentage of variance accounted for by each factor and reliability estimates

Activity-related practices items					
How often do you…	A-MOD†	A-PC	A-PST	A-T/AS†	A-GO†
…set an example for the children by being physically active in front of them?	**0**·**74**				
…play active games with the children (such as playing ball or racing)?	**0**·**71**				
…find fun games that get the children moving?	**0**·**76**				
…play a sport or active game together with the children (and perhaps with other child-care staff)?	**0**·**82**				
…set time aside for active play?	**0**·**62**				
…dance with the children?	**0**·**47**		−0·34		
…play sports games with the children (such as soccer)?	**0**·**77**				
…discipline children for being too active?		**0**·**66**		−0·30	
…tell children they are not (yet) good enough at sports or active games?		**0**·**58**			
…tell children they will get hurt if they play actively?		**0**·**70**			
…reward children for being still?		**0**·**53**			0·36
…not let children play actively for fear of them getting dirty?		**0**·**56**			
…keep children occupied by letting them watch television?			**0**·**83**		
…allow children to watch television for long periods of time?			**0**·**85**		
…allow children to play a lot of video games?			**0**·**53**	0·47	
…say positive things to motivate children to be more active?	0·60			**0**·**21**	
…teach the children new and different ways to be active?	0·55			**0**·**48**	
…teach the children that being active is good for their health?	0·30			**0**·**60**	
…allow children to pick an active game to do together?	0·54			**0**·**59**	
…give the children choices of what physical activities to do?				**0**·**66**	
…go on a walk with the children?					**0**·**87**
…take the children to the park?					**0**·**81**
Percentage of variance accounted for	26·58	10·86	8·10	7·21	5·55
Cronbach’s *α* coefficient	0·85	0·58	0·68	0·75	0·76
Average corrected item–total correlation (for each scale)	0·62	0·35	0·51	0·53	0·61
Range corrected item–total correlation (for the items of each scale)	0·43–0·75	0·24–0·54	0·40–0·59	0·47–0·59	0·61–0·61
Mean	3·66	1·74	1·28	3·57	2·92
sd	0·48	0·48	0·38	0·51	0·79

*n* 116. Factors are labelled as follows: A-MOD, activity-related Modelling; A-PC, activity-related Psychological control; A-PST, activity-related Promote screen time; A-T/AS, activity-related Teaching/Autonomy support; A-GO, Activity-related Going outdoors. Only factor loadings higher than the absolute value of 0·30 are reported. Bold values represent the final factor structure.

† The original ‘encouragement’ factor of the Preschooler Physical Activity Parenting Practices (PPAPP) questionnaire^(^ ^36^ ^)^ was not confirmed, but was divided into three different subscales, namely A-MOD, A-T/AS and A-GO. Sample size used to measure internal consistency estimates: A-MOD, *n* 158; A-PC, *n* 156; A-PST, *n* 124; A-T/AS, *n* 154; A-GO, *n* 158. The single item ‘How often do you have outdoor toys available for the children (e.g. skipping ropes, balls)?’ was not included in the factor analysis, but retained as a single item (mean=4·66, sd=0·51).

The psychometric evaluation of the created five scales is presented in Table 4. Cronbach’s *α* coefficients for the five scales ranged from 0·58 to 0·85. Table 4 also presents average CITC, which suggest adequate consistency of item content within the scales (0·35–0·62). In addition, Table 4 shows the means and standard deviations of the activity-related CFAPQ scales.

### Associations between the CFAPQ scales

Table 5 shows the correlations between the CFAPQ scales. Generally, ‘desirable’ practices were positively correlated with other desirable practices (e.g. food-related Modelling and Encouragement of balance and variety (F-MOD/ENC) showed a strong positive correlation with food-related Teaching about nutrition (F-TN), *r*=0·53, *P*<0·001) and ‘undesirable’ practices were positively correlated with other undesirable practices (e.g. activity-related Psychological control (A-PC) was positively correlated with Promoting screen time (A-PST), *r*=0·24, *P*<0·01). In addition, there were various significant correlations between food-related and activity-related practices, most often when they concerned similar practices although regarding a different behaviour. For instance, food-related Modelling and Encouragement (F-MOD/ENC) was positively associated with activity-related Modelling (A-MOD); *r*=0·33, *P*<0·01) and food-related Teaching about nutrition (F-TN) was positively associated with activity-related Teaching and Autonomy support (A-T/AS; *r*=0·36, *P*<0·001).

**Table 5:** Correlations between the child-care practices as measured by the Child-care Food and Activity Parenting Questionnaire (CFAPQ)

	F-MON	F-MOD/ENC	F-INV/ENV	F-TN	F-PE	F-CC	F-ER/FR	A-MOD	A-PC	A-PST	A-T/AS	A-GO
F-R	**0**·24**	**0**·34***	−0·08	**0**·31***	0·10	−**0**·17*	0·05	0·12	0·12	−0·02	**0**·19*	0·04
F-MON		**0**·24**	0·06	0·15	−**0**·19*	0·12	−0·10	0·12	−0·12	−0·16	**0**·23**	−0·01
F-MOD/ENC			**0**·23**	**0**·53***	0·03	−0·04	−0·09	**0**·33***	−0·06	−**0**·22**	**0**·35**	0·08
F-INV/ENV				0·10	−0·09	0·12	−**0**·19*	0·06	−**0**·21**	−**0**·23**	0·09	0·11
F-TN					0·03	0·00	−0·07	**0**·28***	−0·01	−0·01	**0**·36***	0·12
F-PE						−**0**·18*	**0**·18*	0·00	**0**·40***	0·16	−0·02	**0**·17*
F-CC							−0·04	0·08	0·04	−0·02	0·13	−0·04
F-ER/FR								−0·13	**0**·34***	**0**·20*	−0·07	0·05
A-MOD									−0·02	−**0**·19*	**0**·58***	**0**·19*
A-PC										**0**·24**	−0·07	0·07
A-PST											−0·12	0·05
A-T/AS												0·08

F-R, food-related Restriction; F-MON, food-related Monitoring; F-MOD/ENC, food-related Modelling/Encourage balance and variety; F-INV/ENV, food-related Involvement/Environment; F-TN, food-related Teaching about nutrition; F-PE, food-related Pressure to eat; F-CC, food-related Child control; F-ER/FR, food-related Emotion regulation/Food as reward; A-MOD, activity-related Modelling; A-PC, activity-related Psychological control; A-PST, activity-related Promote screen time; A-T/AS; activity-related Teaching/Autonomy support; A-GO, activity-related Going outdoors. Bold values represent significant correlations. *P<0·05, **P<0·01, ***P<0·001.

### Associations between the CFAPQ and background characteristics

The online supplementary material, Supplemental Table 5 shows the final CFAPQ. With regard to correlations between the CFAPQ and child-care staff’s background characteristics, younger child-care staff scored higher on the activity-related scale Going outdoors (A-GO) than older staff (18–25 years, 3·28; 26–35 years, 2·94; 36–45 years, 2·91; 46–55 years, 3·02; 56–65 years, 2·25; *P=*0·008). Compared with child-care staff who did not have children themselves, child-care staff who did have children scored lower on the food-related scale Emotion regulation/Food as reward (F-ER/FR; 1·39 *v*. 1·23 respectively, *P*=0·016), and the activity-related scales Psychological control (A-PC; 1·85 *v*. 1·67, *P*=0·015) and Going outdoors (A-GO; 3·11 *v*. 2·80, *P*=0·015). Child-care staff’s BMI was positively correlated with the item ‘How often do you have outdoor toys available for the children?’ (*r*=0·198, *P*=0·015). Participants’ educational level was not significantly related to any of the CFAPQ scales or single items. The association between participant’s gender and the CFAPQ scales could not be examined due to the limited number of male child-care staff members in the study.

Child-care staff working in a toddler group (2–4 years) scored higher on the activity-related Teaching/Autonomy support (A-T/AS) scale, compared with child-care staff in other age groups (3·73 *v*. 3·51, *P*=0·016). With regard to the scores on the food-related Modelling/Encourage balance and variety (F-MOD/ENC) scale in relation to participants’ experience in the current child-care centre, an inverted U-shaped association was found: participants who had been working at the same child-care centre for 11–15 years scored the highest on this scale. Participants with less or more experience scored lower (≤5 years, 3·61; 6–10 years, 3·64; 11–15 years, 3·76; >15 years, 3·71; *P*=0·006). A similar reversed U-shaped association was found between the Modelling/Encourage balance and variety (F-MOD/ENC) scale and total number of years working in child-care, although the scores were especially low in the participants with less than 5 years of experience in child care (≤5 years, 3·46; 6–10 years, 3·70; 11–15 years, 3·74; >15 years, 3·66; *P*=0·049). There was a negative correlation between the total number of groups in the child-care centre and the activity-related scale Going outdoors (A-GO; *r*=−0·17, *P*=0·036), indicating that staff working in larger child-care centres less often took the children on field trips.

## Discussion

The aim of the current study was to develop and take the first steps to validate a questionnaire for child-care staff to assess food-related and activity-related practices. Based on two validated parenting practices questionnaires, the CFPQ^(^ ^35^ ^)^ and the PPAPP^(^ ^36^ ^)^, and the previous work of Dev and colleagues^(^ ^33^ ^,^ ^34^ ^)^ to translate the CFPQ to the child-care setting, we developed and validated the Dutch version of the CFAPQ. The scales of the final CFAPQ showed sufficient internal consistency and CITC within acceptable ranges.

The CFAPQ consists of sixty-three items (forty food-related items and twenty-three activity-related), divided over twelve scales (seven food-related and five activity-related scales; see online supplementary material, Supplemental Table 3). The CFAPQ scales are to a large extent similar to those of the original CFPQ^(^ ^35^ ^)^ and PPAPP^(^ ^36^ ^)^ scales. As regards the food-related scales, four CFAPQ scales were in line with the original CFPQ scales: Monitoring, Teaching about nutrition, Pressure to eat and Child control. The three other CFAPQ scales each combined two original CFPQ scales: Encourage balance and variety/Modelling, Environment/Involvement and Emotion regulation/Food as a reward. Regarding the activity-related items, two scales were the same as the original PPAPP scales: Psychological control and Promote screen time. The other items regarding encouragement were distributed over three scales: Modelling of physical activity, Teaching/Autonomy support of physical activity and Going outdoors.

The CFAPQ was developed based on existing parenting practices questionnaires for several reasons. First of all, the role of child-care staff at child care is very similar to parents’ behaviour at home; with the increasing use of child care, child-care staff are becoming increasingly responsible for children’s development during their early years^(^ ^47^ ^)^. As the parenting literature is much more advanced in this area, research in the child-care setting can, or, in our opinion, should, learn and benefit from this. There is, however, another advantage of developing instruments for the child-care setting in line with parenting instruments: it allows for comparison between both settings. In line with an ecological view on environmental influences on children’s energy balance-related behaviours, attunement between the child-care setting and the home setting might be very important^(^ ^48^ ^)^. Studies regarding general child development have for instance shown that parents and child-care staff often have different child-rearing attitudes, values, attitudes and practices^(^ ^49^ ^,^ ^50^ ^)^, and that such inconsistencies have negative effects on children’s well-being^(^ ^50^ ^,^ ^51^ ^)^. The same might be true for children’s food-related and activity-related behaviours. Qualitative studies have repeatedly shown the impact that parents and child-care providers have beyond their own setting, influencing each other’s practices as well as children’s healthy energy balance-related behaviours in the other setting (e.g. references 52–56). This stresses the importance of parent–child-care partnerships. However, quantitative research regarding the importance of continuity between home and child care is lacking^(^ ^48^ ^)^. To be able to examine this, instruments are needed that can be used in both settings^(^ ^48^ ^)^. If different instruments are used to assess practices in the child-care setting than those used in the home setting, any difference between both settings might be caused by methodological flaws, instead of reflecting actual discontinuity. The development of the CFAPQ in line with parenting practices questionnaires allows for comparison of food-related and activity-related practices between the home and child-care settings. There were numerous correlations among the child-care practices scales of the CFAPQ, including cross-behavioural associations between food-related and activity-related practices.

There were various associations between the CFAPQ scales and background characteristics of the child-care staff. Compared with child-care staff who did have children themselves, child-care staff who did not have children more often used undesirable practices: they more often used food to regulate emotions or as a reward, and they more often used psychological control to regulate children’s physical activity. A previous study by Dev and colleagues showed that child-care staff without children more often used pressure to eat^(^ ^34^ ^)^. These findings indicate that experience with children, including experience with staff’s own children, might be an important predictor of positive food-related practices. In line with this, we found that experience within the child-care setting was associated with the use of food-related modelling and encouragement to eat healthily. However, Dev *et al*.^(^ ^34^ ^)^ did not find such an association between years of experience and any of the food-related practices.

Participants’ educational level was not significantly related to any of the CFAPQ scales, which contrasts with the findings of Dev *et al*. in centre-based child care^(^ ^34^ ^)^ and with Brann in family day care^(^ ^54^ ^)^, both reporting more use of pressure to eat by lower educated staff. However, these previous studies compared college graduates with non-college graduates^(^ ^34^ ^,^ ^57^ ^)^, while the current study compared between three similar child-care staff educations, all at intermediate vocational education level. The differences between the levels might have been too small to detect any differences in relation to the scales. In addition, Dev *et al*. linked a number of other characteristics of child-care staff to the practices they use, which were not included in the current study. These included non-white ethnicity, authoritarian feeding style and whether the child-care staff member was trying to lose weight him-/herself, which were all linked to controlling practices^(^ ^34^ ^)^. More research is needed to confirm these predictors, as well as to examine additional predictors. Research regarding food-related parenting practices has for instance shown that the child’s temperament is associated with the practices used by the parents (e.g. reference 16). This might also be the case in the child-care setting. Furthermore, research is needed to examine predictors of activity-related practices, as, to our knowledge, no previous studies reported about this.

The current study had several limitations that need to be acknowledged. First, the sample size was relatively limited, with a sample size of 178 child-care staff members. Moreover, 1028 child-care centres were invited to participate in the study, indicating a maximum response rate of 17·3 % (presuming each participating child-care worker was from a different child-care centre). This low response rate might reflect a selection bias, limiting the generalizability of our findings. Further research in larger samples is thus necessary. Specifically, caution is warranted when interpreting the subgroup analyses, as these analyses are potentially underpowered. Second, it is not known in how many child-care centres these 178 child-care staff members were employed. Child-care centres were approached randomly and asked to inform their employees about the study. We did not register at which child-care centre the participants were working. It was therefore not possible to correct for a potential multilevel structure of the data. If more than one child-care worker participated per centre, these child-care workers would be more alike than child-care workers from different centres, thus potentially explaining part of the associations found in the current study. Third, the associations between the practices and background characteristics were examined using bivariate analyses, thus not taking any potential confounding into account. Fourth, distributions of the answers to some of the items were very skewed and/or had limited variability. This might indicate social desirability bias. Strong points of the current study included building on validated parenting practices instruments^(^ ^35^ ^,^ ^36^ ^)^ and previous research in the child-care setting^(^ ^33^ ^,^ ^34^ ^)^. In addition, the current study combined a qualitative in-depth pre-test with a quantitative pilot test. However, for further validation of the CFAPQ, studies examining the concurrent and predictive value of the questionnaire would be advisable. A good next step would be to examine associations between the CFAPQ scales and other staff characteristics such as physical activity and nutrition-related training and experience. Furthermore, future studies should look at possibilities to reduce the number of items, as the questionnaire is still quite lengthy at this point.

## Conclusion

In conclusion, based on the results of the current study, the CFAPQ seems to be a valid questionnaire to assess child-care staff’s energy balance-related practices, although more research is needed to confirm its validity. The CFAPQ can be used in studies to gain more insight into the use of energy balance-related practices in the child-care setting, predictors of the use of these practices and, perhaps most important, the effect that the practices have on children’s behaviour. Such studies are urgently needed to gain insight into the role the child-care setting can play in obesity prevention and to inform future interventions in the child-care setting.

## Supplementary material

For supplementary material accompanying this paper visit https://doi.org/10.1017/S1368980015003444. click here to view supplementary material
