# Supplementary material for: The Child-care Food and Activity Practices Questionnaire (CFAPQ): development and first validation steps
Source: Public Health Nutr. 2015 Dec 4;19(11):1964–75. doi: 10.1017/S1368980015003444 (PMC4990721; doi:10.1017/S1368980015003444)
Supplement: Supplementary file 1 [file S1368980015003444sup001.doc]

**Supplementary material**

**Supplemental Table 1:** Item conversion Comprehensive Feeding Practices Questionnaire (CFPQ)^(35)^ to child-care setting

| **Original question** | **Question translated to child-care setting** |
| --- | --- |
| ***Child control*** |  |
| Do you let your child eat whatever s/he wants? | Do you let the children eat whatever they want? |
| At dinner, do you let this child choose the foods s/he wants from what is served? | At meals, do you let the children choose the foods they want from what is served? (for example, choose the bread toppings during lunch). |
| If this child does not like what is being served, do you make something else? | If the children don’t like the food that is being served, do you make something else? |
| Do you allow this child to eat snacks whenever s/he wants? | Do you allow the children to eat snacks whenever they want? |
| Do you allow this child to leave the table when s/he is full, even if your family is not done eating? | Do you allow the children to leave the table when they are full, even when the other children are not done eating? |
| ***Emotion regulation*** |  |
| When this child gets fussy, is giving him/her something to eat the *first* thing you do? | When a child gets fussy, is giving him/her something to eat the *first* thing you do? |
| Do you give this child something to eat or drink if s/he is upset even if you think s/he is not hungry? | Do you give a child something to eat or drink if s/he is upset, even if you think s/he is not hungry? |
| ***Encourage balance and variety*** |  |
| Do you encourage this child to eat healthy foods before unhealthy ones? | Do you encourage the children to eat healthy foods before unhealthy ones? |
| I encourage my child to try new foods. | I encourage the children to try new foods. |
| I tell my child that healthy food tastes good. | I tell the children that healthy food tastes good. |
| I encourage my child to eat a variety of foods. | I encourage the children to eat a variety of foods. |
| ***Environment*** |  |
| Most of the food I keep in the house is healthy | Most of the food at the child-care centre is healthy. |
| I keep a lot of snack food (potato chips, Doritos, cheese puffs) in my house. **R** | There are a lot of snack foods present in the child-care centre (for example, crackers, potato chips, cheese puffs). **R** |
| A variety of healthy foods are available to my child at each meal served at home. | A variety of healthy foods are available to the children at each meal served at the child-care centre. |
| I keep a lot of sweets (candy, ice cream, cake, pies, pastries) in my house). **R** | There are a lot of sweets present at the child-care centre (for example, cookies, candy, ice cream). **R** |
|  |  |
| **Original question** | **Question translated to child-care setting** |
| ***Food as reward*** |  |
| I offer sweets (candy, ice cream, cake, pastries) to my child as a reward for good behaviour. | I offer sweets to the children as a reward for good behaviour (for example, cookies, candy, ice cream). |
| I withhold sweets/dessert from my child in response to bad behaviour. | I withhold sweets from the children in response to bad behaviour. |
| I offer my child his/her favourite foods in exchange for good behaviour. | I offer the children their favourite foods in exchange for good behaviour. |
| ***Involvement*** |  |
| I involve my child in planning family meals. | *Not applicable for the child-care setting^a^* |
| I allow my child to help prepare family meals. | I allow the children to help prepare meals (for example, set the table, prepare sandwiches, etc.). |
| I encourage my child to participate in grocery shopping. | *Not applicable for the child-care setting^a^* |
| ***Modelling*** |  |
| I model healthy eating for my child by eating healthy foods myself. | I model healthy eating for the children by eating healthy foods myself. |
| I try to eat healthy foods in front of my child, even if they are not my favourite. | I try to eat healthy foods in front of the children, even if they are not my favourite. |
| I try to show enthusiasm about eating healthy foods. | I try to show enthusiasm about eating healthy foods. |
| I show my child how much I enjoy eating healthy foods. | I show the children how much I enjoy eating healthy foods. |
| ***Monitoring*** |  |
| How much do you keep track of the sweets (candy, ice cream, cake, pies, pastries) that your child eats? | How much do you keep track of the sweets that the children eat (for example, candy, ice cream, cookies)? |
| How much do you keep track of the snack food (potato chips, Doritos, cheese puffs) that your child eats? | How much do you keep track of the snack food that the children eat (for example, salty crackers, potato chips, cheese puffs)? |
| How much do you keep track of the high-fat foods that you child eats? | How much do you keep track of the high-fat foods that the children eat (for example, cheese, sausage, cookies) |
| How much do you keep track of the sugary drinks (soda/pop, kool-aid) this child drinks? | How much do you keep track of the sugary drinks that the children drink (for example, lemonade, chocolate milk, fruit drink)? |
| ***Pressure to eat*** |  |
| My child should always eat all of the food on his/her plate. | The children should always eat all of the food on their plate. |
| If my child says, “I’m not hungry,” I try to get him/her to eat anyway. | If a child says, “I’m not hungry,” I try to get him/her to eat anyway. |
| If my child eats only a small helping, I try to get him/her to eat more. | If a child eats only a small helping, I try to get him/her to eat more. |
| When he/she says he/she is finished eating, I try to get my child to eat one more (two more, etc.) bites of food. | When a child says that he/she is finished eating, I try to get the child to eat another bite of food. |
|  |  |
| **Original question** | **Question translated to child-care setting** |
| ***Restriction for health*** |  |
| If I did not guide or regulate my child’s eating, s/he would eat too much of his/her favourite foods. | If I did not guide or regulate the children’s eating, they would eat too much of their favourite foods. |
| If I did not guide or regulate my child’s eating, he/she would eat too many junk foods. | If I did not guide or regulate the children’s eating, they would eat too many junk foods. |
| I have to be sure that my child does not eat too much of his/her favourite foods. | I have to be sure that the children do not eat too much of their favourite products. |
| I have to be sure that my child does not eat too many sweets (candy, ice cream, cake or pastries). | I have to be sure that the children do not eat too many sweets (for example, candy, ice cream, cookies or pastries). |
| ***Restriction*** ***for weight control*** |  |
| I have to be sure that my child does not eat too many high-fat foods. | I have to be sure that the children do not eat too many high-fat foods (for example, cheese, sausage, cookies). |
| I encourage my child to eat less so he/she won’t get fat. | *Not applicable for the child-care setting^b^* |
| I give my child small helpings at meals to control his/her weight. | *Not applicable for the child-care setting^b^* |
| If my child eats more than usual at one meal, I try to restrict his/her eating at the next meal. | If a child eats more than usual at a one meal, I try to restrict his/her eating at the next meal. |
| I restrict the food my child eats that might make him/her fat. | *Not applicable for the child-care setting^b^*. |
| There are certain foods my child shouldn’t eat because they will make him/her fat. | *Not applicable for the child-care setting^b^* |
| I don’t allow my child to eat between meals because I don’t want him/her to get fat. | *Not applicable for the child-care setting^b^* |
| I often put my child on a diet to control his/her weight. | *Not applicable for the child-care setting^a^* |
| ***Teaching about nutrition*** |  |
| I discuss with my child why it’s important to eat healthy foods. | I discuss with the children why it’s important to eat healthy foods. |
| I discuss with my child the nutritional value of foods. | I discuss with the children the nutritional value of foods. |
| I tell my child what to eat and what not to eat without explanation. **R** | I tell the children what to eat and what not to eat without explanation. **R** |

*Note:* ^a^ Based on expert opinion. ^b^ Based on qualitative pre-test among child-care staff. **R** = reverse coded.

**Supplemental Table 2:** Item conversion Preschooler Physical Activity Parenting Practices (PPAPP) questionnaire of O’Connor and colleagues^(36)^ to child-care setting

| **Original Question** | **Question translated to child-care setting** |
| --- | --- |
| ***Encouragement*** |  |
| How often do you set an example for your child by exercising in front of him/her? | How often do you set an example for the children by being physically active in front of them? |
| How often do you play active games with your child (such as playing ball or racing)? | How often do you play active games with the children (such as playing ball or racing)? |
| How often do you go on a walk with your child? | How often do you go on a walk with the children? |
| How often do you say positive things to motivate your child to be more active? | How often do you say positive things to motivate children to be more active? |
| How often do you play a sport or active game together as a family? | How often do you play a sport or active game together with the children (and perhaps with other child-care staff)? |
| How often do you give your child choices of what physical activities to do? | How often do you give the children choices of what physical activities to do? |
| How often do you allow your child to pick an active game to do together? | How often do you allow children to pick an active game to do together? |
| How often do you dance with your child? | How often do you dance with the children? |
| How often do you play sports games with your child (such as soccer or baseball)? | How often do you play sports games with the children (such as soccer)? |
| How often do you teach your child that being active is good for his/her health? | How often do you teach the children that being active is good for their health? |
| How often do you take your child to the park? | How often do you take the children to the park or forest? |
| How often do you teach your child new and different ways to be active? | How often do you teach the children new and different ways to be active? |
| How often do you take your child to sport practice or game in which he/she is enrolled? | *Not applicable for the child-care setting^a^* |
| How often do you find age appropriate games that get your child moving? | How often do you find fun games that get the children moving? |
| How often do you set time aside for active play? | How often do you set time aside for active play? |
| ***Single items (encouragement)*** |  |
| How often do you not register your child for sports or dance due to lack of money? **R** | *Not applicable for the child-care setting^a^* |
| How often do you have outdoor toys available for your child? | How often do you have outdoor toys available for the children (for example skipping ropes, balls)? |
| ***Promote screen time*** |  |
| How often do you allow your child to watch TV for long periods of time? | How often do you allow children to watch TV for long periods of time? |
| How often do you allow your child to play a lot of videogames? | How often do you allow children to play a lot of videogames? |
| How often do you keep your child occupied by letting him/her watch TV? | How often do you keep children occupied by letting them watch TV? |
| **Original Question** | **Question translated to child-care setting** |
| ***Promote inactivity*** |  |
| How often do you carry your child because he/she does not want to walk | *Not applicable for the child-care setting^a^* |
| How often do you drive your child, when it was easy to walk? | *Not applicable for the child-care setting^a^* |
| How often do you push your child in a stroller instead of letting him/her walk? | *Not applicable for the child-care setting^a^* |
| ***Psychological control*** |  |
| How often do you not let your child play actively for fear of him/her getting dirty? | How often do you not let children play actively for fear of them getting dirty? |
| How often do you tell your child he/she is not good enough at sports or active games? | How often do you tell children they are not (yet) good enough at sports or active games? |
| How often do you tell your child he/she will get hurt if he/she plays actively? | How often do you tell children they will get hurt if they play actively? |
| How often do you discipline your child for being too active? | How often do you discipline children for being too active? |
| How often do you reward your child for being still? | How often do you reward children for being still? |
| ***Restriction for safety concern*** |  |
| How often do you not let your child play outside because you are worried about traffic? | *Not applicable for the child-care setting^b^* |
| How often do you not let your child play outside because you are worried about crime? | *Not applicable for the child-care setting^b^* |
| How often do you not let your child play outside because you are worried about strangers? | *Not applicable for the child-care setting^b^* |
| How often do you let your child go outside to play around your home? **R** | *Not applicable for the child-care setting^b^* |

*Note:* ^a^ Based on expert opinion. ^b^ Based on qualitative pre-test among child-care staff. **R** = reverse coded.

**Supplemental Table 3:** Percentages and means of the individual food-related items, according to the original CFPQ scales

|  | **Frequency (%)** | | | | | | | | | **Mean (sd)** |
| --- | --- | --- | --- | --- | --- | --- | --- | --- | --- | --- |
| **Item** | *Never* | *Rarely* | | *Sometimes* | | *Mostly* | | *Always* | |  |
| ***Child control*** |  |  | |  | |  | |  | |  |
| Do you let the children eat whatever they want? | 12.2% | 19.9% | | 49.4% | | 13.5% | | 5.1% | | 2.8 (1.0) |
| At meals, do you let the children choose the foods they want from what is served? (for example, choose the bread toppings during lunch). | 1.3% | 1.3% | | 10.0% | | 40.0% | | 47.3% | | 4.3 (0.8) |
| If the children don’t like the food that is being served, do you make something else? | 16.3% | 36.6% | | 37.9% | | 7.2 | | 2.0% | | 2.4 (0.9) |
| Do you allow the children to eat snacks whenever they want? | 69.9% | 23.7% | | 5.1% | | 1.3% | | 0.0% | | 1.4 (0.6) |
| Do you allow the children to leave the table when they are full, even when the other children are not done eating? | 57.9% | 24.5% | | 10.7% | | 4.4% | | 2.5% | | 1.7 (1.0) |
| ***Emotion regulation*** |  |  | |  | |  | |  | |  |
| When a child gets fussy, is giving him/her something to eat the *first* thing you do? | 60.8% | 32.3% | | 6.3% | | 0.0% | | 0.6% | | 1.5 (0.7) |
| Do you give a child something to eat or drink if s/he is upset, even if you think s/he is not hungry? | 72.3% | 19.5% | | 8.2% | | 0.0% | | 0.0% | | 1.4 (0.6) |
| ***Monitoring*** |  |  | |  | |  | |  | |  |
| How much do you keep track of the sweets that the children eat (for example, candy, ice cream, cookies)? | 2.9% | 4.3% | | 7.2% | | 36.2% | | 49.3% | | 4.3 (1.0) |
| How much do you keep track of the snack food that the children eat (for example, salty crackers, potato chips, cheese puffs)? | 3.1% | 5.5% | | 6.3% | | 33.9% | | 51.2% | | 4.2 (1.0) |
| How much do you keep track of the high-fat foods that the children eat (for example, cheese, sausage, cookies) | 2.7% | 2.7% | | 11.5% | | 36.5% | | 46.6% | | 4.2 (0.9) |
| How much do you keep track of the sugary drinks that the children drink (for example, lemonade, chocolate milk, fruit drink)? | 2.0% | 3.3% | | 10.0% | | 40.7% | | 44.0% | | 4.2 (0.9) |
| ***Encourage balance and variety*** |  |  | |  | |  | |  | |  |
| Do you encourage the children to eat healthy foods before unhealthy ones? | 6.0% | 5.3% | | 8.6% | | 15.2% | | 64.9% | | 4.3 (1.2) |
|  |  |  | |  | |  | |  | |  |
|  | **Frequency (%)** | | | | | | | | | **Mean (sd)** |
| **Item** | *Disagree* | | *Slightly disagree* | | *Neutral* | | *Slightly agree* | | *Agree* |  |
| ***Encourage balance and variety***  I encourage the children to try new foods. | 0.6% | | 0.6% | | 4.4% | | 30.6% | | 63.8% | 4.6 (0.7) |
| I tell the children that healthy food tastes good. | 1.9% | | 3.1% | | 20.0% | | 30.0% | | 45.0% | 4.1 (1.0) |
| I encourage the children to eat a variety of foods. | 0.6% | | 0.6% | | 13.8% | | 35.8% | | 49.1% | 4.3 (0.8) |
| ***Environment*** |  | |  | |  | |  | |  |  |
| Most of the food at the child-care centre is healthy. | 1.9% | | 5.6% | | 9.9% | | 31.7% | | 50.9% | 4.2 (1.0) |
| There are a lot of snack foods present in the child-care centre (for example, crackers, potato chips, cheese puffs). **R** | 60.9% | | 24.2% | | 7.5% | | 4.3% | | 3.1% | 1.7 (1.0) |
| A variety of healthy foods are available to the children at each meal served at the child-care centre. | 0.0% | | 5.0% | | 8.1% | | 24.2% | | 62.7% | 4.5 (0.8) |
| There are a lot of sweets present at the child-care centre (for example, cookies, candy, ice cream). **R** | 67.9% | | 17.0% | | 9.4% | | 1.9% | | 3.8% | 1.6 (1.0) |
|  |  | |  | |  | |  | |  |  |
| ***Food as reward*** |  | |  | |  | |  | |  |  |
| I offer sweets to the children as a reward for good behaviour (for example, cookies, candy, ice cream). | 91.3% | | 4.3% | | 2.5% | | 1.2% | | 0.6% | 1.2 (0.6) |
| I withhold sweets from the children in response to bad behaviour. | 86.8% | | 3.8% | | 7.5% | | 0.6% | | 1.3% | 1.3 (0.7) |
| I offer the children their favourite foods in exchange for good behaviour. | 88.2% | | 8.1% | | 3.1% | | 0.6% | | 0.0% | 1.2 (0.5) |
|  |  | |  | |  | |  | |  |  |
| ***Involvement*** |  | |  | |  | |  | |  |  |
| I allow the children to help prepare meals (for example, set the table, prepare sandwiches, etc.). | 1.2% | | 0.0% | | 7.5% | | 28.0% | | 63.4% | 4.5 (0.7) |
| ***Modelling*** |  | |  | |  | |  | |  |  |
| I model healthy eating for the children by eating healthy foods myself. | 0.6% | | 1.9% | | 5.7% | | 29.6% | | 62.3% | 4.5 (0.7) |
| I try to eat healthy foods in front of the children, even if they are not my favourite. | 6.3% | | 12.6% | | 8.8% | | 35.8% | | 36.5% | 3.8 (1.2) |
| I try to show enthusiasm about eating healthy foods. | 0.0% | | 1.9% | | 8.2% | | 25.8% | | 64.2% | 4.5 (0.7) |
| I show the children how much I enjoy eating healthy foods. | 3.1% | | 5.0% | | 20.1% | | 34.0% | | 37.7% | 4.0 (1.0) |
|  |  | |  | |  | |  | |  |  |
|  | **Frequency (%)** | | | | | | | | | **Mean (sd)** |
| **Item** | *Disagree* | | *Slightly disagree* | | *Neutral* | | *Slightly agree* | | *Agree* |  |
| ***Pressure*** |  | |  | |  | |  | |  |  |
| The children should always eat all of the food on their plate. | 26.7% | | 25.5% | | 20.5% | | 23.6% | | 3.7% | 2.5 (1.2) |
| If a child says, “I’m not hungry,” I try to get him/her to eat anyway. | 8.1% | | 13.8% | | 28.1% | | 38.1% | | 11.9% | 3.3 (1.1) |
| If a child eats only a small helping, I try to get him/her to eat more. | 12.6% | | 19.5% | | 35.2% | | 29.6% | | 3.1% | 2.9 (1.1) |
| When a child says that he/she is finished eating, I try to get the child to eat another bite of food. | 23.3% | | 25.8% | | 30.2% | | 18.2% | | 2.5% | 2.5 (1.1) |
|  |  | |  | |  | |  | |  |  |
| ***Restriction for health*** |  | |  | |  | |  | |  |  |
| If I did not guide or regulate the children’s eating, they would eat too much of their favourite foods. | 5.0% | | 8.1% | | 16.1% | | 45.3% | | 25.5% | 3.8 (1.1) |
| If I did not guide or regulate the children’s eating, they would eat too many junk foods. | 25.6% | | 13.1% | | 29.4% | | 22.5% | | 9.4% | 2.8 (1.3) |
| I have to be sure that the children do not eat too much of their favourite products. | 11.9% | | 20.8% | | 42.8% | | 22.6% | | 1.9% | 2.8 (1.0) |
| I have to be sure that the children do not eat too many sweets (for example, candy, ice cream, cookies or pastries). | 3.1% | | 5.0% | | 19.5% | | 30.8% | | 41.5% | 4.0 (1.0) |
| ***Restriction*** ***for weight control*** |  | |  | |  | |  | |  |  |
| I have to be sure that the children do not eat too many high-fat foods (for example, cheese, sausage, cookies). | 0.6% | | 5.0% | | 15.5% | | 31.1% | | 47.8% | 4.2 (0.9) |
| If a child eats more than usual at a one meal, I try to restrict his/her eating at the next meal. | 20.0% | | 28.8% | | 30.0% | | 14.4% | | 6.9% | 2.6 (1.2) |
| ***Teaching about nutrition*** |  | |  | |  | |  | |  |  |
| I discuss with the children why it’s important to eat healthy foods. | 2.5% | | 6.9% | | 28.8% | | 33.8% | | 28.1% | 3.8 (1.0) |
| I discuss with the children the nutritional value of foods. | 33.1% | | 12.5% | | 22.5% | | 26.9% | | 5.0% | 2.6 (1.3) |
| I tell the children what to eat and what not to eat without explanation. **R** | 55.3% | | 25.2% | | 16.4% | | 2.5% | | 0.6% | 1.7 (0.9) |

**R** = reverse coded in the final scales. Original unreversed answers are provided in this table. Sd= standard deviation.

**Supplemental Table 4:** Percentages and means of the individual activity-related items , according to the original PPAPP scales

|  | **Frequency (%)** | | | | | | | | | **Mean (sd)** |
| --- | --- | --- | --- | --- | --- | --- | --- | --- | --- | --- |
| **Item** | *Never* | *Rarely* | | *Sometimes* | | *Often* | | *Always* | |  |
| ***Encouragement*** |  |  | |  | |  | |  | |  |
| How often do you set an example for the children by being physically active in front of them? | 0.6% | 1.1% | | 32.2% | | 59.2% | | 6.2% | | 3.7 (0.6) |
| How often do you play active games with the children (such as playing ball or racing)? | 0.0% | 1.7% | | 33.1% | | 60.0% | | 5.1% | | 3.7 (0.6) |
| How often do you go on a walk with the children? | 2.5% | 19.6% | | 46.6% | | 27.0% | | 4.3% | | 3.1 (0.9) |
| How often do you say positive things to motivate children to be more active? | 0.0% | 0.0% | | 16.3% | | 69.9% | | 13.9% | | 4.0 (0.6) |
| How often do you play a sport or active game together with the children (and perhaps with other child-care staff)? | 0.6% | 4.9% | | 43.9% | | 47.6% | | 3.0% | | 3.5 (0.7) |
| How often do you give the children choices of what physical activities to do? | 0.0% | 10.6% | | 40.4% | | 42.2% | | 6.8% | | 3.5 (0.8) |
| How often do you allow children to pick an active game to do together? | 0.0% | 5.8% | | 53.2% | | 39.1% | | 1.9% | | 3.4 (0.6) |
| How often do you dance with the children? | 0.6% | 2.5% | | 38.7% | | 50.3% | | 8.0% | | 3.6 (0.7) |
| How often do you play sports games with the children (such as soccer)? | 0.0% | 3.1% | | 46.9% | | 46.3% | | 3.7% | | 3.5 (0.6) |
| How often do you teach the children that being active is good for their health? | 3.2% | 8.3% | | 26.1% | | 44.6% | | 17.8% | | 3.7 (1.0) |
| How often do you take the children to the park or forest? | 4.9% | 35.2% | | 40.1% | | 18.5% | | 1.2% | | 2.8 (0.9) |
| How often do you teach the children new and different ways to be active? | 0.6% | 3.6% | | 58.4% | | 35.5% | | 1.8% | | 3.3 (0.6) |
| How often do you find fun games that get the children moving? | 0.0% | 1.2% | | 37.3% | | 57.8% | | 3.6% | | 3.6 (0.6) |
| How often do you set time aside for active play? | 0.6% | 0.6% | | 16.7% | | 61.7% | | 20.4% | | 4.0 (0.7) |
| How often do you have outdoor toys available for the children (for example skipping ropes, balls)? | 0.0% | | 0.0% | 1.8% | | 30.7% | | 67.5% | | 4.7 (0.5) |
|  |  | |  | |  |  | |  | |  |
|  | **Frequency (%)** | | | | | | | | | **Mean (sd)** |
| **Question translated to child-care setting** | *Never* | *Rarely* | | *Sometimes* | | *Often* | | *Always* | |  |
| ***Promote screen time*** |  | |  | |  | |  | |  |  |
| How often do you allow children to watch TV for long periods of time? | 77.2% | | 21.4% | | 1.4% | | 0.0% | | 0.0% | 1.2 (0.5) |
| How often do you allow children to play a lot of videogames? | 89.6% | | 8.9% | | 0.8% | | 0.0% | | 0.0% | 1.1 (0.4) |
| How often do you keep children occupied by letting them watch TV? | 54.0% | | 40.1% | | 5.8% | | 0.0% | | 0.0% | 1.5 (0.6) |
| ***Psychological control***  How often do you not let children play actively for fear of them getting dirty? | 68.6% | | 25.6% | | 5.3% | | 0.6% | | 0.0% | 1.4 (0.6) |
| How often do you tell children they are not (yet) good enough at sports or active games? | 76.0% | | 18.6% | | 5.4% | | 0.0% | | 0.0% | 1.3 (0.6) |
| How often do you tell children they will get hurt if they play actively? | 21.6% | | 42.6% | | 28.4% | | 6.2% | | 1.2% | 2.2 (0.9) |
| How often do you discipline children for being too active? | 60.2% | | 30.4% | | 9.3% | | 0.0% | | 0.0% | 1.5 (0.7) |
| How often do you reward children for being still? | 21.4% | | 33.3% | | 34.6% | | 10.7% | | 0.0% | 2.4 (0.9) |

**Supplemental Table 5:** The final version of the Child-care Food and Activity Practices Questionnaire (CFAPQ)

| **Item** | **Scale** | **Answering scale**^a^ |
| --- | --- | --- |
| If I did not guide or regulate the children’s eating, they would eat too many junk foods. | Food-related - Restriction (F-R) | A |
| I have to be sure that the children do not eat too much of their favourite products. | Food-related - Restriction (F-R) | A |
| I have to be sure that the children do not eat too many sweets (for example, candy, ice cream, cookies or pastries). | Food-related - Restriction (F-R) | A |
| I have to be sure that the children do not eat too many high-fat foods (for example, cheese, sausage, cookies). | Food-related - Restriction (F-R) | A |
| If a child eats more than usual at a one meal, I try to restrict his/her eating at the next meal. | Food-related - Restriction (F-R) | A |
| If I did not guide or regulate my child’s eating, s/he would eat too much of his/her favourite foods. | Food-related - Restriction (F-R) | A |
| How much do you keep track of the sweets that the children eat (for example, candy, ice cream, cookies)? | Food-related - Monitoring (F-MON) | B |
| How much do you keep track of the snack food that the children eat (for example, salty crackers, potato chips, cheese puffs)? | Food-related - Monitoring (F-MON) | B |
| How much do you keep track of the high-fat foods that the children eat? (for example, cheese, sausage, cookies) | Food-related - Monitoring (F-MON) | B |
| How much do you keep track of the sugary drinks that the children drink (for example, lemonade, chocolate milk, fruit drink)? | Food-related - Monitoring (F-MON) | B |
| I model healthy eating for the children by eating healthy foods myself. | Food-related – Modelling / Encourage balance and variety (F-MOD/ENC) | A |
| I try to eat healthy foods in front of the children, even if they are not my favourite. | Food-related – Modelling / Encourage balance and variety (F-MOD/ENC) | A |
| I try to show enthusiasm about eating healthy foods. | Food-related – Modelling / Encourage balance and variety (F-MOD/ENC) | A |
| I show the children how much I enjoy eating healthy foods. | Food-related – Modelling / Encourage balance and variety (F-MOD/ENC) | A |
| I encourage the children to try new foods. | Food-related – Modelling / Encourage balance and variety (F-MOD/ENC) | A |
| I tell the children that healthy food tastes good. | Food-related – Modelling / Encourage balance and variety (F-MOD/ENC) | A |
| I encourage the children to eat a variety of foods. | Food-related – Modelling / Encourage balance and variety (F-MOD/ENC) | A |
| I allow the children to help prepare meals (for example, set the table, prepare sandwiches, etc.). | Food-related – Involvement / Environment (F-INV/ENV) | A |
| Most of the food at the child-care centre is healthy. | Food-related – Involvement / Environment (F-INV/ENV) | A |
| There are a lot of snack foods present in the child-care centre (for example, crackers, potato chips, cheese puffs). **R** | Food-related –Involvement / Environment (F-INV/ENV) | A |
| A variety of healthy foods are available to the children at each meal served at the child-care centre. | Food-related – Involvement / Environment (F-INV/ENV) | A |
| There are a lot of sweets present at the child-care centre (for example, cookies, candy, ice cream). **R** | Food-related – Involvement / Environment (F-INV/ENV) | A |
| I discuss with the children why it’s important to eat healthy foods. | Food-related - Teaching about nutrition (F-TN) | A |
| I discuss with the children the nutritional value of foods. | Food-related - Teaching about nutrition (F-TN) | A |
| I tell the children what to eat and what not to eat without explanation. **R** | Food-related - Teaching about nutrition (F-TN) | A |
| The children should always eat all of the food on their plate. | Food-related – Pressure to eat (F-PE) | A |
| If a child says, “I’m not hungry,” I try to get him/her to eat anyway. | Food-related – Pressure to eat (F-PE) | A |
| If a child eats only a small helping, I try to get him/her to eat more. | Food-related – Pressure to eat (F-PE) | A |
| When a child says that he/she is finished eating, I try to get the child to eat another bite of food. | Food-related – Pressure to eat (F-PE) | A |
| Do you let the children eat whatever they want? | Food-related – Child control (F-CC) | B |
| At meals, do you let the children choose the foods they want from what is served? (for example, choose the bread toppings during lunch). | Food-related – Child control (F-CC) | B |
| If the children don’t like the food that is being served, do you make something else? | Food-related – Child control (F-CC) | B |
| Do you allow the children to eat snacks whenever they want? | Food-related – Child control (F-CC) | B |
| Do you allow the children to leave the table when they are full, even when the other children are not done eating? | Food-related – Child control (F-CC) | B |
| When a child gets fussy, is giving him/her something to eat the *first* thing you do? | Food-related – Emotion regulation / Food as reward (F-ER/FR) | B |
| Do you give a child something to eat or drink if s/he is upset, even if you think s/he is not hungry? | Food-related – Emotion regulation / Food as reward (F-ER/FR) | B |
| I offer sweets to the children as a reward for good behaviour (for example, cookies, candy, ice cream). | Food-related – Emotion regulation / Food as reward (F-ER/FR) | A |
| I withhold sweets from the children in response to bad behaviour. | Food-related – Emotion regulation / Food as reward (F-ER/FR) | A |
| I offer the children their favourite foods in exchange for good behaviour. | Food-related – Emotion regulation / Food as reward (F-ER/FR) | A |
| Do you encourage the children to eat healthy foods before unhealthy ones? | *Single item* | B |
| How often do you set an example for the children by being physically active in front of them? | Activity-related – Modelling (A-MOD) | B |
| How often do you play active games with the children (such as playing ball or racing)? | Activity-related – Modelling (A-MOD) | B |
| How often do you find fun games that get the children moving? | Activity-related – Modelling (A-MOD) | B |
| How often do you play a sport or active game together with the children (and perhaps with other child-care staff)? | Activity-related – Modelling (A-MOD) | B |
| How often do you set time aside for active play? | Activity-related – Modelling (A-MOD) | B |
| How often do you dance with the children? | Activity-related – Modelling (A-MOD) | B |
| How often do you play sports games with the children (such as soccer)? | Activity-related – Modelling (A-MOD) | B |
| How often do you discipline children for being too active? | Activity-related – Psychological control (A-PC) | B |
| How often do you tell children they are not (yet) good enough at sports or active games? | Activity-related – Psychological control (A-PC) | B |
| How often do you tell children they will get hurt if they play actively? | Activity-related – Psychological control (A-PC) | B |
| How often do you reward children for being still? | Activity-related – Psychological control (A-PC) | B |
| How often do you not let children play actively for fear of them getting dirty? | Activity-related – Psychological control (A-PC) | B |
| How often do you keep children occupied by letting them watch TV? | Activity-related – Promote screen time (A-PST) | B |
| How often do you allow children to watch TV for long periods of time? | Activity-related – Promote screen time (A-PST) | B |
| How often do you allow children to play a lot of videogames? | Activity-related – Promote screen time (A-PST) | B |
| How often do you say positive things to motivate children to be more active? | Activity-related – Teaching / Autonomy support (A-T/AS) | B |
| How often do you teach the children new and different ways to be active? | Activity-related – Teaching / Autonomy support (A-T/AS) | B |
| How often do you teach the children that being active is good for their health? | Activity-related – Teaching / Autonomy support (A-T/AS) | B |
| How often do you allow children to pick an active game to do together? | Activity-related – Teaching / Autonomy support (A-T/AS) | B |
| How often do you give the children choices of what physical activities to do? | Activity-related – Teaching / Autonomy support (A-T/AS) | B |
| How often do you go on a walk with the children? | Activity-related – Going outdoors (A-GO) | B |
| How often do you take the children to the park? | Activity-related – Going outdoors (A-GO) | B |
| How often do you have outdoor toys available for the children (e.g. skipping ropes, balls)? | *Single item* | B |

*Note:* ^a^ Answering scale A: totally disagree (1), slightly disagree (2), neutral (3), slightly agree (4), totally agree (5). Answering scale B: never (1), rarely (2), sometimes (3), mostly (4), always (5). For use of the questionnaire, the items should be randomised.
